# European biologic training course for type 2 inflammation by EUFOREA in 2024: key facts and lessons learned

**DOI:** 10.3389/falgy.2024.1517122

**Published:** 2024-12-12

**Authors:** D. M. Conti, V. Backer, W. Fokkens, P. Gevaert, A. Peters, G. K. Scadding, I. Pavord, S. Lau, M. Wechsler, X. Bertels, G. Liva, M. Doulaptsi, E. Prokopakis, P. W. Hellings

**Affiliations:** ^1^ Allergy and Clinical Immunology Research Unit, Department of Microbiology and Immunology, KU Leuven, Leuven, Belgium; ^2^ Escuela de Doctorado UAM, Centro de Estudios de Posgrado, Universidad Autónoma de Madrid, Calle Francisco Tomás y Valiente, Madrid, Spain; ^3^ The European Forum for Research and Education in Allergy and Airway Diseases Scientific Expert Team Members, Brussels, Belgium; ^4^ Department of Otorhinolaryngology, Head & Neck Surgery, and Audiology, Rigshospitalet, Copenhagen University, Copenhagen, Denmark; ^5^ Department of Otorhinolarynogology and Head/Neck Surgery, Amsterdam University Medical Centres, Location AMC, University of Amsterdam, Amsterdam, Netherlands; ^6^ Upper Airways Research Laboratory, Department of Head and Skin, Ghent University, Ghent, Belgium; ^7^ Department of Medicine, Division of Allergy and Immunology, Northwestern University Feinberg School of Medicine, Chicago, IL, United States; ^8^ Department of Allergy & Rhinology, Royal National ENT Hospital, London, United Kingdom; ^9^ Division of Immunity and Infection, University College, London, United Kingdom; ^10^ Respiratory Medicine, NIHR Oxford Biomedical Research Centre, Nuffield Department of Medicine, University of Oxford, Oxford, United Kingdom; ^11^ Department of Pediatric Respiratory Medicine, Immunology and Critical Care Medicine, Charité Universitätsmedizin Berlin, Berlin, Germany; ^12^ Department of Psychiatry and Behavioral Sciences, Duke University Medical Center, Durham, NC, United States; ^13^ Division of Biostatistics and Bioinformatics, National Jewish Health, Denver, CO, United States; ^14^ Department of Epidemiology, Erasmus MC, Rotterdam, Netherlands; ^15^ Department of Otorhinolaryngology, University of Crete, School of Medicine, Heraklion, Greece; ^16^ KU Leuven Department of Microbiology and Immunology, Allergy and Clinical Immunology Research Unit, Leuven, Belgium; ^17^ Clinical Department of Otorhinolaryngology, Head and Neck Surgery, University Hospitals Leuven, Leuven, Belgium

**Keywords:** EUFOREA, asthma, rhinosinusitis, type 2 inflammation, training course, biologics

## Abstract

The European Forum for Research and Education in Allergy and Airways diseases (EUFOREA) organized the first European Biologic Training Course (EBTC) in Brussels on 1st March 2024. The aim of this hybrid EBTC including both face-to-face and web-based participation was to address the educational needs of physicians dealing with asthma and Chronic Rhinosinusitis with Nasal Polyps (CRSwNP) on the clinically relevant aspects of diagnosing and treatment with biologics. EUFOREA is an international non-for-profit organization forming an alliance of all stakeholders dedicated to reducing the prevalence and burden of chronic respiratory diseases through the implementation of optimal patient care via educational, research, and advocacy activities. The inclusive and multidisciplinary approach of EUFOREA was reflected in faculty coming from the paediatric, allergology, pulmonology, and Ear, Nose and Throat (ENT) speciality and from different continents, with more than 250 participants from over 30 countries in the first EBTC. The current report provides a comprehensive overview of key statements made by the faculty of the EBTC 2024, especially focusing on patient selection for a biologic drug, the communication with patients, the onset of biological treatment and the follow-up in routine clinical practice.

## Introduction

Chronic airways and allergic diseases are widespread health concerns that affect millions of people worldwide, not least in westernized society where they have reached epidemic proportions (1). Conditions driven by type 2 inflammation such as asthma and CRSwNP can cause significant morbidity, reduced quality of life, and increased healthcare costs (2). The prevalence of type 2 driven inflammatory conditions has risen globally. Several of these diseases start in childhood or adolescence presenting opportunities for timely treatment with the aim of achieving disease control and even remission. Therefore, researchers, clinicians and patients need to better understand type 2 inflammation, to address both major unmet needs and arrest or retard disease progress before an irreversible chronic status is reached (3–5).

**Figure 1:**
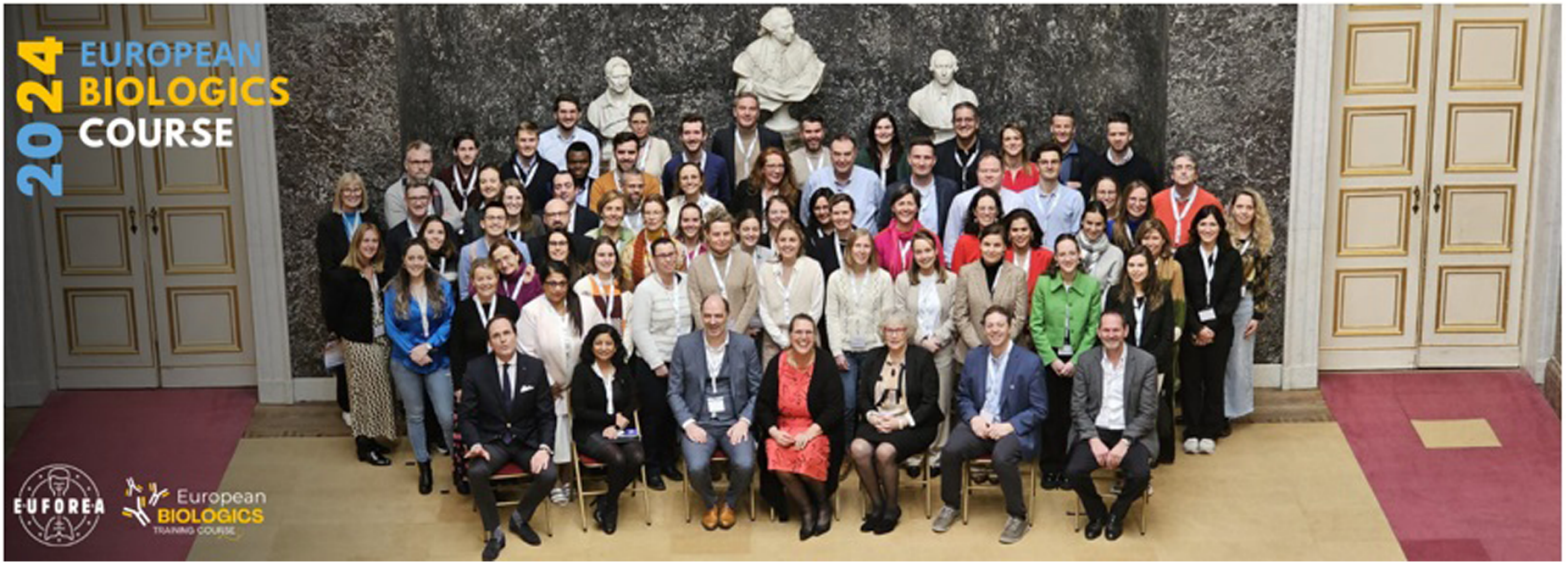
EBTC group photo.

EUFOREA is an international non-for-profit organization founded in 2015 on the suggestion of the European Commissioner of Health Vytenis Andriukaitis, forming an alliance of multiple stakeholders dedicated to reducing the prevalence and burden of chronic respiratory diseases through the implementation of optimal patient care via educational, research, and advocacy activities. Based on EUFOREA's core values of inclusivity and innovation, the *EBTC* was organized with the ambition to bring to the attention of a large and global audience state-of-the art knowledge on monoclonal antibodies (mAbs) used in treatment of Type 2 diseases with focus on practical considerations on biologics for asthma and CRSwNP and guidance in the choice and follow-up of biologic treatment. The collaboration between specialists and specialists-in-training in pulmonology, allergology, ENT and paediatrics reflects the ambition of EUFOREA of being inclusive and multidisciplinary (Figure 1).

The *EBTC* reunited well-recognized global experts to present their lectures aiming to reach the following key learning objectives: (1) Practical considerations on biologics for asthma and CRSwNP and both, including biomarkers and treatable traits, and (2) Guidance in the choice and follow-up of a biologic for asthma and CRSwNP.

The *EBTC* increased and highlighted the need for a multidisciplinary approach to T2-driven diseases (6) and their complications focusing on Biologics. Overall, the discussions underscored the need for increased attention and resources in research, education, and advocacy to address unmet needs. The full content of the *EBTC* is available on the EUFOREA website under the e-Academy section.

## Which patients are suitable candidates for biologic therapy? Asthma vs. CRSwNP

Currently, there are six approved biologics for asthma (omalizumab, mepolizumab, reslizumab, benralizumab, dupilumab, and tezepelumab), with three also approved for CRSwNP (omalizumab, mepolizumab and dupilumab). However, a considerable amount of time has passed since the initial therapeutic attempt in patients, and research leading to the approval of new biologics for asthma and CRSwNP (7). Initial high expectations and suboptimal study designs of the early study led to industry hesitancy. CRSwNP, in particular, was not regarded as a compelling indication for a long time. The early research focused on anti-IgE with the possibility to treat severe allergic asthma with omalizumab (8, 9) and also CRSwNP (2). Shortly after, anti-bodies towards IL-5 and the receptor for IL-5, were developed (8, 9) first as intravenous administration and further developed for subcutaneous injection. Subsequent research elucidated the role of biologic treatment in Asthma vs. CRSwNP (10–13). While most biologics targeting eosinophils have proven effective in asthma, it is now known that only antibodies against IL-5, such as mepolizumab, also have a significant impact on CRSwNP (14–16). However, targeting type 2 inflammation further upstream by blocking the IL-4 receptor with dupilumab results in a greater effect in CRSwNP (14–16). The evidence of efficacy of these drugs becomes increasingly clear (17, 18), which has led to its implementation in the present era, facilitated by reimbursement policies adopted by health systems that can afford it.

The criteria for the indication of biologics are diverse and depend on each healthcare system, its reimbursement policy and the consideration of comorbidities associated with the underlying disease present in each case. In this sense, each patient must be assessed in their individual context. However, the common denominator is patients who have not responded to standard therapies and need systemic corticosteroids should be offered alternatives such as biologics (19–21). The knowledge from asthma treatment with mAbs has been that some patients with eosinophilic asthma respond on some drugs, and other patients do not. Here is a lack of biomarker driven selection of the patients possibly responding on a specific drug. The typical patients with severe asthma are patients with 2 or more exacerbations, in need of high dose inhaled corticosteroids (ICS) and courses of systemic steroid, in spite of double or triple maintenance therapy. These patients have been evaluated with the focus of treatable traits (22) to eliminate any possible comorbidity pushing to the uncontrolled disease activity. In the case of CRSwNP, the typical profile is that of a patient who has not achieved an adequate response to appropriate medical treatment (AMT), or to one or two short courses of oral corticosteroids, or to surgical intervention followed by AMT (21, 23, 24). In this context, it is necessary to pose a number of questions in order to assist medical judgement:

– Are there biomarkers indicating Type 2 disease in asthma and/or CRSwNP?
– Is the patient exhibiting disease control or is the patient suffering from an uncontrolled disease?
– Does the patient have any comorbidities, or have we identified any treatable traits (22) during a systematic assessment?

If all treatable treats have been addressed and the patient continues to suffer from uncontrolled disease despite appropriate medical treatment, then this may be an optimal candidate for treatment with monoclonal antibodies or other relevant biological drugs.

In light of the aforementioned considerations, EUFOREA has put a set of criteria for the indication of biologics in patients with CRSwNP (25, 26). This noteworthy academic endeavour has established a foundation upon which numerous health systems have subsequently established their reimbursement criteria. The current criteria should be to shift the focus from damage-based biomarkers to activity-based biomarkers. In practice, this implies the identification and treatment of patients presenting with early manifestations of disease, with the objective of achieving a high impact with treatment (25, 26). In already severe cases, the positive impact is expected to be smaller (25, 26). Comparatively, this would be the case for severe asthma or Chronic Obstructive Pulmonary Disease (COPD).

GINA has proven to be a failure-based treatment escalator. These guidelines do not consider any activity markers but only severity markers indicated by increased symptoms (27, 28). The practical and clinical consequence of this approach is that it forces professionals and patients to wait too long before implementing more complex therapeutic schemes, allowing damage to accumulate in the meantime. This is akin to looking backwards rather than forwards. This might explain why the new biologics have not been less effective in patients with severe disease, but have been successful in those with less advanced forms of asthma (27, 28).

In asthmatic patients, FeNO and blood eosinophil count are useful markers of disease activity (27, 28). Both have positive predictive value for developing more severe forms of asthma and exacerbations (29). Furthermore, they have cumulative predictive value when both are altered (29). However, this presents a therapeutic opportunity. Firstly, it is evident that the response of these patients to biologics is highly favourable in both cases (30, 31). Secondly, it has been demonstrated that the earlier these patients are treated, the more favourable the response and the lower the risk of progression in severity and exacerbation or the development of comorbidities (32, 33).

## Expected outcomes of biological care: asthma vs. CRSwNP

All patients with asthma and chronic rhinosinusitis (CRS) share a common burden of disease to a greater or lesser extent (34). EUFOREA has compiled a list of the most important aspects of their disease burden, and has found similarities not only in the aspects themselves, but also in the experiences and expectations of patients regarding their therapeutic journey (34). Another common aspect was the inequalities of care (35). These deficiencies encompass shortcomings in the healthcare system, a lack of optimal therapeutic approaches, a dearth of state-of-the-art knowledge among healthcare professionals, and a lack of knowledge among patients (36). In light of the aforementioned considerations, EUFOREA advocates for a gradual approach towards asthma or CRSwNP patients. There is a plethora of therapeutic options available, and the approach must be based on a correct diagnosis, contextualised to the individual patient. The proposed therapy should be agreed with the patient and should increase in therapeutic complexity only when the progression of the disease demands it, after reconsideration of the underlying diagnosis and the suspicion and search for associated comorbidities (34–36).

The arsenal of biologics available for asthma patients is extensive, with anti-IgE, anti-IL-5, anti-IL-4/IL-13 and anti-TSLP currently in use. The right biologic should be applied to the right patient at the right stage of the disease, with the potential to reduce exacerbations, reduce systemic steroid and local steroid doses and their side effects, improve the quality of life of the patient, to reach remission/disease modification to prevent asthma over the long term or to even reach a cure (27–33). Biologics should be considered for patients with poorly controlled asthma, as defined by the presence of asthma symptoms despite the use of ICS and long-acting beta-agonists and/or long-acting muscarine antagonist (ICS/LABA/LAMA), GINA step 4–5, interference with daily activities such as sport and work or sleep, chronic use of oral corticosteroids, and at least two exacerbations in the previous year depending on the recommendation of the specific country (27–33). The attending physician would have good arguments to indicate biologics in any of the above cases. All of the aforementioned considerations should be guided by the principle outlined above. This entails confirming the diagnosis of asthma, optimising therapy (which typically involves ICS/LABA/LAMA), often also montelukast, confirming adherence and inhalation technique, and addressing complicating comorbidities (such as sinus disease, reflux, aspiration and/or OSA). Once asthma has been confirmed, therapy is optimised, adherence/technique is optimised and comorbidities are addressed, biologics can then be considered (27–33). In cases of allergic asthma, allergen immunotherapy (AIT) should be considered while treating with biologic drugs which lead to a better disease control which is allowing to perform AIT towards for example house dust mites, this could lead to a higher likelihood of disease remission of asthma (37).

The case of CRSwNP is analogous, as it has a range of therapeutic options and an increasing number of biologics that have been approved for use (26). When evaluating a patient with uncontrolled CRSwNP, it is essential to consider the possible causes of lack of control, including disease-related factors (such as smoking, occupational irritants or environmental triggers) and treatment-related factors (lack of symptom-oriented treatment, incorrect dose or route of therapeutic administration, or suboptimal treatment), patient factors (non-compliance, incorrect use of medication or unreliable patient) and/or diagnosis-related factors (structural pathology or incorrect diagnosis) (38), all of which is similar in asthma.

## How to start a biologic in daily practice

What to tell a patient about biologics has not been subject to specific training or education yet. Patients often have specific questions like expected outcomes, or concerns such as the risk of infection, specific situations such as pregnancy or breastfeeding, or direct concerns about adverse effects. Based on the experience gained, EUFOREA proposes the key points in Table 1. Once the general consultation has been completed, the therapeutic options should be presented for discussion with the patient to enable the patients to on a supervised foundation to take the decision together with the health care staff (Table 2). Aspects to be discussed that should not be underestimated include: the number of times the patient should receive treatments, number of times to come at the hospital, possible comorbidities related to the primary diagnosis, possible pregnancy status or planned pregnancy in the short term (39), increased risk of anaphylaxis (40), risk of malignancy (41), associated infections (42) and other adverse effects.

**Table 1:** What to inform a patient about biologics.

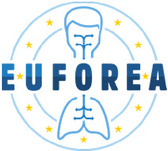 What to inform a patient about biologics	
CRSwNP	Asthma
Benefits	
Reduction of symptom severity with improvement of QoL	
Reduced medication need	
Mostly likely arrest the progress of disease (disease modification)	
Decreased need for revision surgery	Reduction of exacerbations
---	Improvement of lung function
---	Improved physical activity
Considerations	
Health-economic considerations	
Monitoring of adverse events	
Long-term duration of treatment	

**Table 2:** Latest therapeutic agents with their age-related licence.

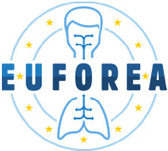 Biologics and its applications on asthma and CRSwNP					
Molecule	Target	Action mechanism	Application	Age indications	Indications
Omalizumab	IgE	Inhibiting the binding of the IgE antibody to IgE receptors	SC/2–4 wk	≥6 years	Severe eosinophilic asthma/CRSwNP
Mepolizumab	IL-5	Reduction of blood eosinophil levels	SC/4 wk	≥6 years	Severe eosinophilic asthma/CRSwNP
Reslizumab	IL-5	Reduction of eosinophil activation, survival, and recruitment	IV/4 wk	≥18 years (data for ≥12 years)	Severe eosinophilic asthma
Benralizumab	IL-5R*α*	Antibody-dependent triggering of cellular cytotoxicity	SC/4–8 wk	≥18 years (data for ≥12 years)	Severe eosinophilic asthma
Dupilumab	IL-4Rα/IL-13	Inhibition of IL-4Rα/IL-13 signalling	SC/2 wk	≥6 years for severe type 2 asthma ≥6 months for severe atopic dermatitis	Severe eosinophilic asthma/CRSwNP
Tezepelumab	TSLP	Reduction of downstream activation of multiple inflammatory pathways, including those involving eosinophils, basophils and Th2 cells.	SC/2 wk	≥12 years	Severe eosinophilic asthma

Arrangement of reimbursement files is not a standardised process due to the aforementioned differences in the criteria set by different health systems. In asthma, the experience dates back to around 2010, where treatment with biologics were adopted into daily clinic. As for now many hundreds of patients have started treatment and remission have been seen in some patients whereas disease control has been found in many patients over the world, at least in the western societies. The disease markers of important in asthma care, would be evaluation of adherence, inhaler technique, calculation of ACQ or ACQ or similar, Lung function, FeNO, and often blood or sputum eosinophilic count as well. This seems to be a minimum when evaluating asthma patients in treatment with biologic drugs.

When referring to CRSwNP, despite the differences around the world, the common denominator is the outcome/criteria of EUFOREA and EPOS (43). Many initiatives have attempted to standardise the basic and essential data required. The EUFOREA recommendation is to collect the following basic and essential data: Bilateral Endoscopic Nasal Polyp Score (NPS), Sino-Nasal Outcome Test (SNOT-22), Lund-Mackay CT Scoring System, Nasal Congestion Score (NCS), Total Rhinosinusitis Symptoms Score (TSS) and Olfactory Dysfunction evaluated using a smell test.

There are intrinsic considerations and complexities when administering a biologic, such as the angle of administration and injection site (Table 3). The abdomen or quadriceps skin is always preferred. Although very rare, a severe allergic reaction can occur and should be anticipated (44). Special cases such as pregnancy and lactation have been addressed in previous work and do not appear to have a negative influence (45). Parasitosis is another special situation, and although it has not been shown to be induced or predisposed by biologics, it should be treated before starting this line of therapy (46). Nor has there been any reported adverse effect on the interaction between biologics and attenuated vaccines (47).

**Table 3:** How to carry out the first injection and what to prepare.

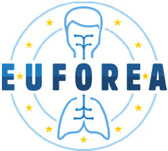 How to carry out the first injection and what to prepare
Assessment and definition of disease phenotype and biomarkers
Assessment of co-morbidities
Treat the treatable traits possible before starting biologic drugs
Diagnosis of Type-2 disease
Select the right mAbs for the right patient (tailored medication)
Safety
Anaphylaxis—Recommendations are like other injections
Knowledge among the staff for injection (epi-pen or other as well as mAbs)
Education of the patients to perform self-administration
Other treatments, like influenza injection
Parasitic possibilities (worms before first injection treat, tropical travelling and postpone mAbs injections

Finally, in terms of recommended follow-up, the authors recommend the following schedule of follow-up clinic visits at one, three and six months and one year after the first injection. Finally, with regard to suggested follow-up times, the authors recommend the usual schedule of consultations after the first month, after the third or fourth month, after the sixth month and one year after the first injection. With regard to the questions that should guide follow-up, this group advocates the following:

– What was the reason for intensification and how has it changed?
– Did the patient have an exacerbation?
– Did the patient respond?
– Did the patient achieve remission?

Negative answers to these questions should prompt the treating physician to reconsider the underlying diagnosis, suspect the presence of new or previously unrecognised associated comorbidities, and reconsider the therapeutic strategy.

## Practical implications related to the use of biologics

The traditional approach to a patient with asthma, whereby a diagnosis is made including systematic assessment with the focus of treatable traits and a standardised treatment is prescribed, must be abandoned. It is now proposed that the same treatment will not always be applicable to all patients, just as one size does not fit all. It is therefore necessary to identify biomarkers and treatable traits that should be assessed in the context of the individual patient (23, 37, 48, 49, 50). This is one of the criteria for selecting the right therapeutic choice for the right patient, including biologics. Furthermore, it can inform the physician's the likelihood of response to these therapeutics (Table 4). A practical example is the use of blood eosinophil counts (51) or FeNO (52) as biomarkers to predict which patients are more likely to suffer asthma exacerbations in the near future or not. Similarly, both have positive predictive value for understanding which patients will have the greatest therapeutic benefit, particularly in terms of reduced corticosteroid requirements, with biologics (53, 54). This has orderly being described in the recent published pocket book of asthma (37). Contextualising the disease within the individual patient's circumstances and considering the aforementioned factors enables a more nuanced analysis of which biologic is most appropriate for each case (Table 5).

**Table 4:** Factors associated with response to biologics.

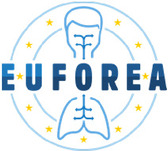 Factors associated with response to biologics		
Increased	Neutral	Reduced
Eosinophils in blood and NPs	Allergy	Bronchodilator reversibility
FeNO	Symptom scores	Reduced FEV1% predicted
Frequency of asthma attacks	Sex	Obesity
Use of OCS	Serum IgE	Airway damage and infection
Adult onset		

**Table 5:** Factors favouring individual biologics.

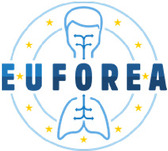 Factors favoring individual biologics for asthma		
Anti-IL-5	Anti-IL-4 R	Anti-TSLP
Blood eosinophils >500	FeNO >50 ppb	Multiple raised biomarkers
Attack frequency	Atopic dermatitis	Airway Hyperresponsiveness
OCS dependence	CRSwNP	Allergy
Nasal polyposis	Early onset	Early onset
Adult onset	Allergy	Impaired lung function
Pregnancy	Impaired lung function	Type-2 low asthma

It has been demonstrated that nearly 90% of CRSwNP are related to type 2 inflammation in the western societies (55, 56), which has led to the development of biologic options. These have been shown to greatly benefit patients with CRSwNP by reducing outcome parameters like NPS, NCS, VAS, and SNOT-22 (57), and increasing the sense of smell, and furthermore, reducing the need for steroids or surgical interventions (57). This is where the academic work of EUFOREA is of particular value, as it presents its pocket guides (23, 37, 48, 49, 50) and establishes the criteria for the consideration and inclusion of biologics in patients with CRSwNP (21).

There is no consensus on the optimal timing for follow-up after the initiation of biologic therapy. This group proposes a set of criteria for evaluating efficacy, as expressed in Table 6. Ultimately, health systems are required to monitor, evaluate and follow up on these patients, and this is also a criterion.

**Table 6:** How to evaluate efficacy in biologics treatment.

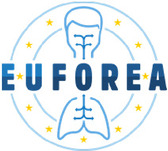 How to evaluate efficacy in biologics treatment	
Asthma	CRSwNP
Asthma control with ACQ < 1.0 or ACT > 15	SNOT-22 score ≤ 12
Reduction in exacerbation	Normalization of sense of smell
Normal or better level of lung function	50% reduction in nasal polyp size or a reduction in nasal polyp score of at least 1
Low, lower or no systemic steroids	

The achievement of therapeutic success can be defined by three parameters: therapeutic response/improvement, remission (clinical or biological) and disease modification or cure. However, the subsequent steps to be taken once this status has been achieved are less clear. The options are to continue with the current therapeutic(s), to stop them, to stop only some of them or to implement the taper of them. In the latter case, it has been observed that a significant proportion of patients continue to respond well to the treatment, and in these patients, it would not be necessary to revert to a more complex therapeutic approach (58, 59). As it is well known, that for some of the biologic drugs they have sustained effect after termination of treatment and others might not (60).

Conversely, considerations must be taken into account when measuring response, as outlined in Table 7. The current standard therapeutic goal should be remission. Remission is debated very intensively in both Asthma and CRSwNP, as is it remission under continuation of treatment, or remission when the treatment with biologic drugs could be terminated and disease control are maintained, or is it cure of disease. The strict criteria not only restrict the number of patients who can be treated, but also result in the delay of biologics being introduced until more severe stages of the disease have been reached. This reduces the chance of remission or even cure that could occur if the patient were to receive them (61–63).

**Table 7:** Assessing response.

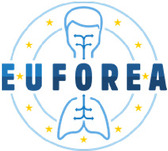 Assessing response in asthma		
Super responder patients (40%)	Responder patients (40%)	Non-responder patients (20%)
Maintenance OCS—off maintenance OCS and no exacerbation OR remains on small dose of OCS due to HPA axis suppression only	Maintenance OCS—>50% reduction in OCS maintenance dose with >50% reduction in exacerbation rate	All other combinations
For those not on OCS—no exacerbation	For those not on OCS—>50% reduction in exacerbation rate	

The existing literature on the mental health implications of biologic treatment is inconclusive. While there are reports on the relationship between depression and biologic treatment response in other diseases, such as rheumatoid arthritis (64), the available evidence also includes case reports of patients who, as a consequence of experiencing positive results with such therapy, report improvements in their general context and their perception of the disease (65). Further research, including more reliable measures, is needed to define this aspect.

## What will the future of biological care bring?

In the field of pulmonology, the identification of biomarkers will enable the identification of patients who may benefit from a specific biologic (27, 28). The positive therapeutic effect of several biologics has been repeatedly demonstrated, compelling us to pursue disease remission as a goal. They have been shown to result in up to an 80% reduction in exacerbations as well as use of systemic steroid treatment and a substantial improvement in lung function (27, 28). These results are not only remarkable but also have the potential to significantly enhance the quality of life for patients. It is imperative to emphasise the necessity of implementing these therapeutic strategies, hitherto reserved for severe cases, at the earliest stages of the disease. This will not only save the patient time but will prevent the disease from progressing towards severe forms, which are associated with significant damage and a substantial burden on the health system (24, 62, 63).

The outlook for patients with CRSwNP is encouraging. According to the most recent data, 15% of patients respond positively to nasal corticosteroid-only regimens (24). For those who receive the surgical option, a revision rate of 15 to 20% has been estimated (66, 67). Some reports have illustrated lack of control in 40% of patients with CRSwNP at 3 years after sinus surgery (68). Another report 12 years after sinus surgery has shown that 47.4% was uncontrolled, 26.30% was partially controlled and 26.30% was controlled (69). If we would use the EUFOREA definition on Remission, we could state that 26.30% of the patients reached a long-term remission or even cure 12 years after sinus surgery in the pre-biologic era (26). A review of the literature reveals that recent therapeutic options for severe CRSwNP include drug-eluting stents, other/improved nasal spray applications and new biologics (70). There is an increasing body of evidence suggesting that remission and cure may be achievable in patients with severe CRSwNP (71), even in cases with associated comorbidities (72). The proportion of patients who remain uncontrolled despite the available options is decreasing and has reached record lows (73). The field of biologics is undergoing a shift towards a greater focus on Type 2 targets, while also exploring and proposing strategies for non-type targets (73).

Many patients with lower airway disease suffer of CRS as well, it has been shown that around 50% of patients with asthma have from mild to severe CRSwNP which need to be treated accordingly (68, 74, 75). Former studies have shown a better asthma control is achieved when patients with double disease (asthma and CRSwNP) have treatment with either nasal corticosteroids (37) or FESS surgery (23, 37, 48, 49, 50). Furthermore, between 50%–70% of patients with severe CRSwNP also have asthma (37) and it also seems as well treated asthma affect the nasal disease control, although data are less obvious, probably due to lack of monitoring system for severity of the disease and change in severity, whereas asthma monitoring tools like the Asthma Control Questionnaire are widely used in most asthma centres. Systematic assessment with the focus of treatable traits is needed either in a collaborative setting or a combined clinic setting (6). The clinicians need to secure the easiness for systematic assessment for all patients, whether the entrance is in an asthma clinic, an allergy clinic or an ENT clinic. This might need a patients Coordinator, like used in cancer unites, to ensure a safe route and evaluation for all patients.

## Summary

The EBTC offered a unique perspective on biologics for type 2 inflammatory diseases, with the focus of diagnose of diseases, systematic assessment, treatable traits, optimal care, prevention and remission. The current landscape of biologics research is becoming increasingly diverse and promising. The new treatment paradigm for patients with asthma and/or CRSwNP is and will be to achieve remission of the disease and cure. EUFOREA will continue to contribute its efforts to positively contribute to the quality of life of these patients.

## Data Availability

The original contributions presented in the study are included in the article/Supplementary Material, further inquiries can be directed to the corresponding author.
